# The effect of LysaKare infusion on serum potassium levels in patients with gastroenteropancreatic neuroendocrine tumours eligible for treatment with [ ^177^Lu]Lu‐DOTA‐TATE: A post‐authorisation safety study

**DOI:** 10.1111/jne.70172

**Published:** 2026-04-13

**Authors:** Johannes Hofland, Chiara Maria Grana, Martin O. Weickert, Andrew R. Moore, Tahir Shah, Vineet Prakash, Agnieszka Kolasińska‐Ćwikła, Wouter W. de Herder, Francesca Spada, Lingfei Xu, Ramon Fite, Yuan Wu, Jarosław B. Ćwikła

**Affiliations:** ^1^ Department of Internal Medicine, Section of Endocrinology, ENETS Center of Excellence Erasmus MC Cancer Institute Rotterdam The Netherlands; ^2^ Radiometabolic Therapy Unit, Division of Nuclear Medicine European Institute of Oncology (IEO), IRCCS Milan Italy; ^3^ The ARDEN NET Centre, ENETS Centre of Excellence University Hospitals Coventry and Warwickshire NHS Trust Coventry UK; ^4^ Liverpool University Hospitals NHS Foundation Trust Liverpool UK; ^5^ University Hospital Birmingham NHS Foundation Trust Birmingham UK; ^6^ Royal Surrey County NHS Foundation Trust Surrey UK; ^7^ Maria Sklodowska‐Curie National Research Institute of Oncology Warsaw Poland; ^8^ Division of Gastrointestinal Medical Oncology and Neuroendocrine Tumors European Institute of Oncology (IEO), IRCCS Milan Italy; ^9^ Novartis Pharmaceuticals Corp, East Hanover New Jersey USA; ^10^ Novartis Pharma AG Barcelona Spain; ^11^ Novartis Pharma AG Basel Switzerland; ^12^ University of Warmia and Mazury Olsztyn Poland

**Keywords:** ^177^Lu‐DOTATATE, Hyperkalaemia, LysaKare

## Abstract

A post‐authorisation safety study (PASS) was conducted to assess the impact of LysaKare (2.5% Lysine‐Arginine solution) on serum potassium concentrations and its safety/tolerability profile in patients with gastroenteropancreatic neuroendocrine tumours (GEP‐NET) eligible for ^177^Lu‐DOTATATE treatment. In a phase IV, multicentre, single‐arm, open‐label PASS, adults with somatostatin receptor‐positive GEP‐NET who were eligible for ^177^Lu‐DOTATATE treatment received 1000 mL of LysaKare (monotherapy) intravenously over 4 h. The primary endpoint was the change in serum potassium levels over the course of 24 h. Secondary endpoints included the incidence of LysaKare‐related adverse events (AEs) and changes in laboratory parameters. Forty‐one patients were treated (median age, 57 years; 92.7% White and 7.3% Black; 53.7% male) and 40 completed post‐treatment follow‐up. Mean (standard deviation [SD]) serum potassium was 4.33 (0.397) mmol/L pre‐dose, increasing by 0.60 (0.666) mmol/L after 4 h (time to maximum change, range, 2–24 h) before decreasing to around the pre‐dose level: the mean (SD) increase after 24 h was 0.07 (0.396) mmol/L. Other electrolytes and blood gas components mostly showed transient changes that lasted ~24 h. A general trend of transient metabolic acidosis was also observed based on laboratory values. Serum potassium grade increased from baseline in 41.5% of patients. By comparison, five patients (12.2%) had a treatment‐related AE of hyperkalaemia (grade 3, *n* = 1); all resolved within 24 h, either without treatment (*n* = 3) or following intravenous furosemide (*n* = 2). There were no serious AEs and no AEs leading to treatment discontinuation/interruption. This PASS identified no new safety signals attributable to LysaKare.

Trial registration: EudraCT, 2019‐004073‐76. Registered 20/08/2020.

## 1 INTRODUCTION

The radioligand therapy (RLT) [^177^Lu]Lu‐DOTA‐TATE—referred to as lutetium (^177^Lu) oxodotreotide in the European Union (hereafter ^177^Lu‐DOTATATE)—is indicated for the treatment of adult patients with unresectable or metastatic, progressive, well‐differentiated (grades 1 and 2), somatostatin receptor‐positive gastroenteropancreatic neuroendocrine tumours (GEP‐NET) in the European Union.^1^ Additionally, ^177^Lu‐DOTATATE is approved by the US Food and Drug Administration for use in paediatric patients aged ≥12 years with somatostatin receptor‐positive GEP‐NET, based on results from the NETTER‐P trial.^2^, ^3^ ^177^Lu‐DOTATATE has also been shown to prolong progression‐free survival in patients aged ≥15 years with newly diagnosed, advanced, well‐differentiated (higher grade 2 and grade 3, Ki67 ≥10% and ≤55%), somatostatin receptor‐positive GEP‐NET within the NETTER‐2 study.^4^ LysaKare, used for the reduction of renal radiation exposure during RLT with ^177^Lu‐DOTATATE in adults, is a 2.5% Lysine‐Arginine solution that was approved by the European Medicines Agency (EMA) in July 2019.^5^ Similar solutions containing 2.5% L‐lysine and 2.5% L‐arginine have been used for more than 10 years in Europe and the existing scientific evidence for these contributed to the approval of LysaKare.^5^, ^6^, ^7^, ^8^, ^9^

Treatment with amino acid solutions, including LysaKare, is associated with a risk of hyperkalaemia.^6^, ^7^, ^9^, ^10^ Administration of cationic amino acids (e.g., L‐lysine and L‐arginine) may result in metabolic acidosis, which can lead to hyperkalaemia by shifting potassium from the intracellular to the extracellular compartment^8^, ^11^ and by decreasing potassium secretion and increasing reabsorption in collecting ducts.^8^ Previous studies using lysine‐arginine amino acid solutions have found that increased serum potassium levels are generally transient and typically return to near baseline within 24 h.^6^, ^7^, ^10^

A post‐authorisation safety study (PASS) was required as part of the risk‐management plan following approval from the EMA for marketing authorisation for LysaKare infusion. The primary objective was to assess the effect of LysaKare administration (without co‐administration of ^177^Lu‐DOTATATE) on serum potassium concentrations in patients with GEP‐NET eligible for treatment with ^177^Lu‐DOTATATE. The secondary objective was to characterise the safety and tolerability profile of LysaKare monotherapy in patients with GEP‐NET eligible for treatment with ^177^Lu‐DOTATATE.

## 2 MATERIALS AND METHODS

### 2.1 Study design and population

A prospective, phase IV, multicentre, single‐arm, open‐label PASS was conducted across seven active centres: in the UK (four centres), Italy (one centre), Netherlands (one centre), and Poland (one centre) (EudraCT number: 2019‐004073‐76). Patients were aged ≥18 years at screening, had somatostatin receptor‐positive GEP‐NET, and were eligible for treatment with ^177^Lu‐DOTATATE per the ^177^Lu‐DOTATATE label.^1^ Key exclusion criteria included: pre‐existing hyperkalaemia at screening (>6.0 mmol/L; >5.5 mmol/L in Poland, following a local protocol amendment) if not adequately corrected before LysaKare infusion; instances where ^177^Lu‐DOTATATE was not recommended per the ^177^Lu‐DOTATATE label;^1^ pregnancy or lactation, or a positive pregnancy test result at screening or before LysaKare infusion; a history of hypersensitivity to the active substances in LysaKare; and ^177^Lu‐DOTATATE treatment prior to screening or RLT treatment scheduled within 7 days of LysaKare treatment.

The study treatment was a 1000‐mL solution of LysaKare (2.5% Lys‐Arg solution for infusion), administered intravenously over a 4‐h period, at an infusion rate of 250 mL/h. Only one infusion was administered in the treatment phase. Before infusion, patients received an intravenous antiemetic, per clinical practice.^5^ ^177^Lu‐DOTATATE was not administered in this study.

### 2.2 Endpoints and assessments

The primary endpoint was change in serum potassium levels at specified time points (2, 4, 6, 8, 12, and 24 h) after LysaKare administration, compared with baseline (0 h). The primary endpoint analysis was performed on patients who had received any volume of LysaKare and had serum potassium measurements prior to starting the infusion and at least once 4–8 h after starting the infusion, which was when the peak potassium level was expected to occur (evaluable set). This analysis was performed after the follow‐up telephone call (48 h post‐infusion) with the last enrolled patient.

Secondary endpoints included incidence of LysaKare‐related adverse events (AEs), changes in vital signs and electrocardiogram (ECG) parameters, and changes in laboratory parameters. Analyses of secondary endpoints were conducted for patients who received any administration of LysaKare (safety set).

The prespecified secondary endpoint ‘changes in laboratory parameters’ included changes from baseline at specified time points (2, 4, 6, 8, 12, and 24 h) following LysaKare administration for potassium levels in the safety set (irrespective of the sample source used after infusion initiation) and other electrolytes (i.e., bicarbonate and chloride) and blood gas parameters (i.e., pH and partial pressure CO_2_). While this secondary analysis included post‐baseline potassium measurements from all sample sources, the primary endpoint was limited to serum potassium measurements as potassium levels measured from any source do not necessarily account for potential haemolysis and thus may underestimate actual serum potassium levels.^12^, ^13^ The analysis of changes in other electrolytes and blood gas parameters enabled assessment of metabolic acidosis development following LysaKare administration.

All AEs were recorded from provision of informed consent until the end of the follow‐up telephone call (48 h post‐infusion). AEs were coded using the Medical Dictionary for Regulatory Activities Version 25.0,^14^ and classified per the Common Terminology Criteria for Adverse Events (CTCAE) Version 5.0.^15^ Vital signs were assessed at 0, 2, 4, 6, 8, 12, and 24 h. ECGs were recorded in triplicate at 0, 4, 8, and 24 h. Samples were collected at 0, 2, 4, 6, 8, 12, and 24 h for serum electrolyte and venous blood gas assessments, and at 0 and 24 h for haematology and serum blood chemistry.

A post‐hoc exploratory analysis was performed (safety set) to stratify post‐baseline potassium increase, that is, worsening from baseline of the CTCAE grade for serum potassium levels (increase), by pre‐treatment estimated glomerular filtration rate (eGFR). Pre‐treatment eGFR was estimated using data from electronic case report forms (serum creatinine, age, and sex, per the 2021 CKD‐EPI Creatinine Equation). The association between post‐baseline potassium increase status and pre‐treatment eGFR score was also assessed.

### 2.3 Statistical analyses

The study aimed to enrol approximately 45 patients to ensure that ≥25 patients were evaluable for the primary endpoint. The sample size was based on the length of the confidence interval of the mean change in serum potassium levels at 4 h (i.e., the time at which the maximum mean increase was expected), calculated using t‐distribution. Based on historical findings—that is, that mean potassium levels increase by 0.9 mmol/L (standard deviation [SD]: 0.3) at 4 h following infusion of a 2.5% Lysine‐Arginine solution^16^—it was initially proposed that the total confidence interval should not exceed 0.2 mmol/L. That is, assuming an SD of 0.3, the distance should not exceed 0.1 mmol/L from the mean to the limit of the confidence interval. It was estimated that this could be achieved with a sample of 38 patients, although the sample size was set at 45 to account for potential patient drop‐out. However, it was ultimately decided that a minimum of 25 evaluable patients was required for the primary endpoint analysis to achieve a confidence interval total length of 0.25 mmol/L.

Concomitant therapy was defined as all interventions (therapeutic treatments and procedures) other than the study treatment that were administered during the treatment period. Concomitant therapy included medications starting on or after the start date of study treatment, or medications starting prior to and continuing after the start date of study treatment.

The study was descriptive in nature and no hypothesis was tested for the study endpoints. For the post‐hoc exploratory analysis exploring the relationship between post‐baseline potassium increase status and pre‐treatment eGFR score, a Pearson correlation coefficient was generated. Otherwise, data were summarised using descriptive statistics. For all statistical analyses, SAS version 9.4 software (SAS Institute; Cary, NC; http://www.sas.com; RRID: SCR_008567) was used.

## 3 RESULTS

### 3.1 Baseline demographics and clinical characteristics

**FIGURE 1:**
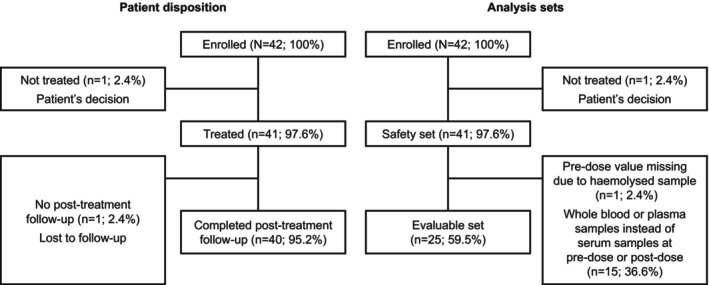
Patient disposition and analysis sets.

Overall, 42 patients were enrolled between 25 January 2021 and 18 November 2023 (Figure 1). In total, 41 patients were treated and 40 completed the post‐treatment follow‐up; one patient was lost to follow‐up (no follow‐up telephone contact).

The median age of all patients was 57 years (range, 34–79 years) (Table 1). Most patients were aged <65 years (68.3%) and White (92.7%). Sex distribution was well balanced. The most common primary tumour sites were the pancreas (41.5%), ileum (26.8%), and midgut (19.5%). Most patients (90.2%) had World Health Organization stage IV GEP‐NET. A similar number of patients had grade 1 (46.3%) and grade 2 (53.7%) GEP‐NET. Overall, 92.7% of patients had at least one prior/concomitant antineoplastic therapy/procedure for the study indication.

**TABLE 1:** Baseline demographic and clinical characteristics (safety set).

Baseline characteristic	All patients (*N* = 41)
Age, years (median, range)	57.0 (34–79)
<65 years, no. (%)	28 (68.3)
≥65 years, no. (%)	13 (31.7)
Sex, no. (%)	
Female	19 (46.3)
Male	22 (53.7)
Race, no. (%)	
White	38 (92.7)
Black	3 (7.3)
BMI, kg/m^2^	*n* = 40
Mean (SD)	26.78 (4.93)
Median (range)	25.47 (18.8–42.3)
Creatinine clearance grade,^a^ no. (%)	
0	37 (90.2)
1	2 (4.9)
2	2 (4.9)
Primary site of cancer, no. (%)	
Pancreas	17 (41.5)
Ileum	11 (26.8)
Midgut	8 (19.5)
Rectum	2 (4.9)
Ascending colon	1 (2.4)
Cecum	1 (2.4)
Jejunum	1 (2.4)
Disease stage, no. (%)	
IIIB	3 (7.3)
IV	37 (90.2)
Not reported	1 (2.4)
NET grade (according to Ki‐67 index), no. (%)	
1 (<3%)	19 (46.3)
2 (3%–20%)	22 (53.7)
3 (>20%)	0
Presence of metastases, no. (%)	
Yes	38 (92.7)
No	3 (7.3)
Number of metastatic sites,^b^ no. (%)	
<3	19 (50.0)
≥3	19 (50.0)
Months since initial diagnosis to study entry	
Median (range)	52.2 (2–212)
Months since most recent recurrence/relapse	*n* = 40
Median (range)	2.1 (0–109)
Patients with ≥1 prior/concomitant antineoplastic therapy/procedure, no. (%)	38 (92.7)
Medications	33 (80.5)
Procedure/surgery	25 (61.0)
Radiotherapy^c^	2 (4.9)

Abbreviations: BMI, body mass index; NET, neuroendocrine tumour; SD, standard deviation.

^a^ Grades based on Common Terminology Criteria for Adverse Events Version 5.0.

^b^ Number of metastatic sites refers to number of unique sites reported per patient (of 38 patients with metastases).

^c^ External beam radiotherapy.

Based on a post‐hoc exploratory analysis, the mean (SD) pre‐treatment eGFR was 89.23 (17.49) mL/min/1.73 m^2^.

### 3.2 Medical history, prior therapies, and concomitant medications (safety set)

The most common ongoing medical conditions were hypertension (17 [41.5%]) and type 2 diabetes mellitus (10 [24.4%]). Two patients (4.9%) had carcinoid syndrome at baseline.

Sixteen patients (39.0%) had received prior medications. The most frequently used prior therapies (>10% of patients) were for diabetes (6 [14.6%]) and lipid‐modifying agents (5 [12.2%]). At baseline, 15 patients (36.6%) had received prior therapies that could have increased potassium levels (angiotensin‐converting enzyme inhibitors [perindopril, ramipril, benazepril, lisinopril], angiotensin receptor blockers [candesartan], nonsteroidal anti‐inflammatory drugs [diclofenac], heparins [enoxaparin, nadroparin], laxatives [potassium chloride], potassium canrenoate). Additionally, seven patients (17.1%) had received prior medications that could have decreased potassium levels (diuretics [hydrochlorothiazide, indapamide], adrenergics [salbutamol], laxatives [potassium chloride]), and all continued these as concomitant medications after study treatment started. One patient had grade 1 hypokalaemia at baseline; this patient had received treatment with indapamide.

All patients (*N* = 41) received concomitant medications during the study. Of note, all patients received medication before treatment to reduce nausea and vomiting, which are common AEs associated with LysaKare. These medications were recorded under multiple anatomical therapeutic chemical classes, most commonly antiemetics and antinauseants; 38 patients (92.7%) received these concomitantly, while one patient (2.4%) received granisetron the day before treatment. Two patients received antiemetics under other classes before treatment: one (2.4%) received cyclizine (‘antihistamines for systemic use’) and one (2.4%) received domperidone (‘drugs for functional gastrointestinal disorders’). Aside from antiemetics and antinauseants, other frequently used concomitant medications were lipid‐modifying agents (12 [29.3%]) and diabetes therapies (11 [26.8%]).

### 3.3 Treatment exposure (safety set)

LysaKare infusion duration and total volume administered were completed as planned for all patients (Supplementary Table 1).

### 3.4 Dose modifications (safety set)

No patients discontinued treatment. Six patients (14.6%) had a brief infusion interruption (range, 5–12 min), unrelated to AEs.

### 3.5 Change in potassium levels

**FIGURE 2:**
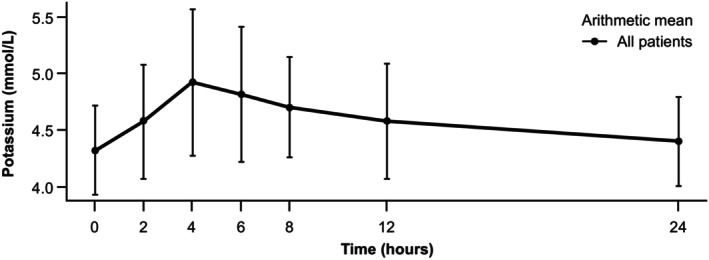
Mean (SD) concentration–time profile for serum potassium results (evaluable set). SD, standard deviation.

Among the 41 patients treated with LysaKare (i.e., the safety set), 25 patients had serum potassium measurements taken before the infusion and 4–8 h after starting the infusion; these patients (i.e., the evaluable set, which met the predefined sample size requirements) were evaluable for the primary endpoint. The remaining 16 patients were not included in the primary analysis, primarily (*n* = 15) due to having potassium measured from whole blood or plasma instead of serum (Figure 1), but were included in the secondary analysis. The primary analysis revealed that serum potassium levels increased transiently and returned to near baseline within 24 h (Figure 2; Table 2). The mean (SD) pre‐dose serum potassium level was 4.33 (0.397) mmol/L. Serum potassium level peaked at 4 h post‐dose, with a mean (SD) absolute change of 0.60 (0.666) mmol/L compared with the pre‐dose level. At 24 h post‐dose, the mean (SD) absolute change from the pre‐dose level was 0.07 (0.396) mmol/L, indicating a return to around the pre‐dose level. The mean (SD) maximum change at any time point was 0.82 (0.617) mmol/L (Table 3). The median time to maximum change was 4.3 h (range, 2–24 h).

**TABLE 2:** Serum potassium (mmol/L) by time point (evaluable set).

	All patients (*N* = 25)	
	Mean (SD)	Median (IQR)
Pre‐dose (*n* = 25)	4.33 (0.397)	4.25 (4.03–4.59)
2‐h assessment (*n* = 23)	4.58 (0.502)	4.60 (4.26–4.76)
Change from baseline	0.25 (0.452)	0.20 (0.00–0.40)
Percent change from baseline	5.99 (10.390)	4.34 (0.00–9.52)
4‐h assessment (*n* = 25)	4.92 (0.648)	4.70 (4.53–5.34)
Change from baseline	0.60 (0.666)	0.70 (0.30–0.83)
Percent change from baseline	14.26 (15.203)	14.49 (7.69–21.05)
6‐h assessment (*n* = 25)	4.82 (0.601)	4.70 (4.47–5.20)
Change from baseline	0.49 (0.602)	0.60 (0.20–0.80)
Percent change from baseline	11.79 (13.695)	13.04 (4.88–20.51)
8‐h assessment (*n* = 25)	4.71 (0.445)	4.60 (4.40–5.00)
Change from baseline	0.38 (0.487)	0.43 (0.20–0.67)
Percent change from baseline	9.28 (11.040)	9.30 (4.88–17.14)
12‐h assessment (*n* = 24)	4.58 (0.514)	4.47 (4.23–4.76)
Change from baseline	0.24 (0.557)	0.18 (−0.03 to 0.47)
Percent change from baseline	6.05 (12.198)	4.16 (−0.54 to 11.58)
24‐h assessment (*n* = 25)	4.40 (0.390)	4.32 (4.11–4.71)
Change from baseline	0.07 (0.396)	0.10 (−0.10 to 0.22)
Percent change from baseline	2.09 (8.877)	2.38 (−2.63 to 4.57)

Abbreviations: IQR, interquartile range; SD, standard deviation.

**TABLE 3:** Maximum change in serum potassium levels (evaluable set).

	All patients (*N* = 25)
Maximum change,^a^ mmol/L	
Mean (SD)	0.82 (0.617)
Median	0.75
Q1–Q3	0.48–1.20
Range	−0.6 to 2.6
Time to maximum change, hours	
Median	4.3
Q1–Q3	4.0–6.0
Range	2–24

Abbreviations: Q, quartile; SD, standard deviation.

^a^ Peak level post‐dose minus pre‐dose level.

**FIGURE 3:**
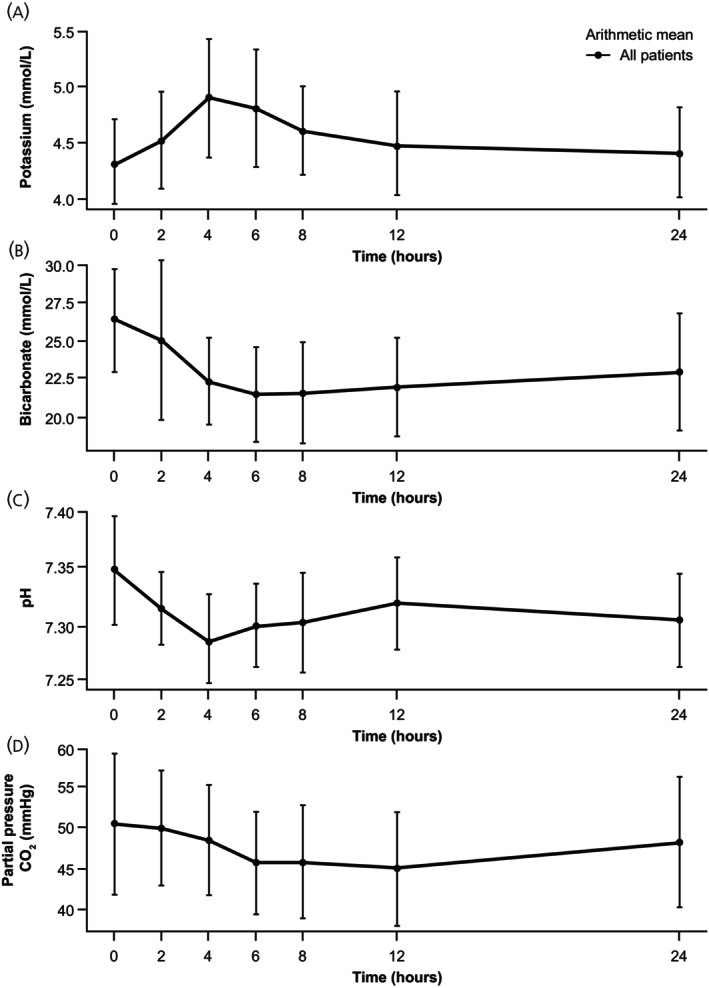
Mean (SD) concentration–time profiles for (A) potassium, (B) bicarbonate, (C) pH, and (D) partial pressure CO_2_, irrespective of the sample source (safety set). SD, standard deviation.

The secondary analysis of all 41 patients with post‐infusion potassium levels (irrespective of sample source) yielded results that were similar to the primary analysis of serum potassium. In the safety set, a peak was observed at 4 h, with a mean (SD) absolute change of 0.57 (0.591) mmol/L from the pre‐dose level; this returned to around the pre‐dose level at 24 h post‐dose, with a mean (SD) absolute change of 0.09 (0.434) mmol/L (Figure 3A). No differences in this trend or in overall changes in potassium levels were observed between males and females (data not reported).

### 3.6 Change in other electrolytes and blood gas parameters (safety set)

**FIGURE 4:**
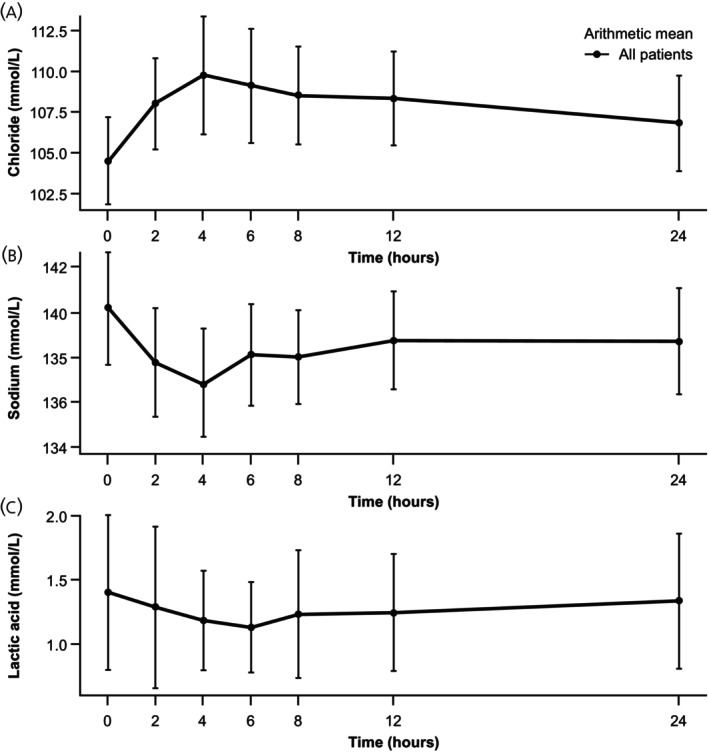
Mean (SD) concentration–time profiles for (A) chloride, (B) sodium, and (C) lactate, irrespective of the sample source (safety set). SD, standard deviation.

Further electrolyte and blood gas component analyses, irrespective of the sample source, were performed on the safety set (Figures 3 and 4). Like potassium, other electrolyte and blood gas parameter levels changed over time, with all except bicarbonate returning to near baseline levels within 24 h. There was a general trend towards metabolic acidosis following treatment, as shown by the decrease in pH and bicarbonate and compensated by the slight decrease in partial pressure CO_2_ (Figure 3B–D); in most cases, this was resolving within 24 h.

### 3.7 Adverse events (safety set)

Eleven patients (26.8%) experienced ≥1 AE during the study (Table 4). There were no serious AEs, no deaths, and no AEs leading to treatment discontinuation/interruption.

**TABLE 4:** AEs by System Organ Class and Preferred Term (safety set).

	All patients (*N* = 41)	
	All grades, no. (%)	Grade ≥3, no. (%)
At least one AE	11 (26.8)	1 (2.4)
Gastrointestinal disorders	5 (12.2)	0
Nausea	2 (4.9)	0
Abdominal pain	1 (2.4)	0
Proctalgia	1 (2.4)	0
Vomiting	1 (2.4)	0
General disorders/administration‐site conditions	1 (2.4)	0
Fatigue	1 (2.4)	0
Injury, poisoning, and procedural complications	1 (2.4)	0
Contusion	1 (2.4)	0
Metabolism and nutrition disorders	5 (12.2)	1 (2.4)
Hyperkalaemia	5 (12.2)	1 (2.4)
Nervous system disorders	2 (4.9)	0
Dizziness	1 (2.4)	0
Headache	1 (2.4)	0
Respiratory, thoracic, and mediastinal disorders	1 (2.4)	0
Respiratory acidosis	1 (2.4)	0

*Note*: Data are the no. (%) of patients with AEs. AEs were classified according to Common Terminology Criteria for Adverse Events Version 5.0.

Abbreviation: AE, adverse event.

The most frequently reported AEs were hyperkalaemia (*n* = 5, 12.2%) and nausea (*n* = 2, 4.9%) (Table 4). Treatment‐related AEs occurred in six patients (14.6%): hyperkalaemia in five patients (12.2%) and vomiting in one patient (2.4%). All AEs were grade 1 (9.8%) or 2 (14.6%), except for one grade 3 AE of hyperkalaemia (i.e., serum potassium >6.0 mmol/L).

Hyperkalaemia was the only AE of special interest and was resolved within 24 h, either without treatment (*n* = 3) or with intravenous diuretic (furosemide) treatment (*n* = 2). Potassium levels in the patient with grade 3 hyperkalaemia were 4.3 mmol/L pre‐dose, increasing to 6.9 mmol/L at 4 h post‐dose and 6.1 mmol/L at 6 h post‐dose, before returning to the normal range (5.1 mmol/L) at 24 h post‐dose. This patient received furosemide (40 mg) intravenously twice daily, and 1 litre of sodium chloride intravenously.

### 3.8 Vital signs and ECG parameters (safety set)

No notable vital sign values were reported. Notable ECG parameters (based on all ECG values) are presented in Table 5. New absolute QT interval corrected for heart rate by Fridericia's formula (QTcF) values of >450 to ≤480 ms were observed in seven patients (17.9%). No QTcF increase >60 ms was reported. From an aggregate perspective, median increase of QTcF from baseline was <3 ms at 4 h. New PR >200 ms was observed in six patients (20.7%). Excluding two patients with notable values in a single ECG assessment, notable mean ECG values (QT, PR, and QTcF) were observed in six patients; none were considered clinically significant or reported as AEs.

**TABLE 5:** Notable ECG values (safety set).

Parameter	Notable criteria	All patients (*N* = 41), *n/m* (%)
QTcF, ms	Increase >30 to ≤60 ms	7/41 (17.1)
	Increase >60 ms	0/41 (0)
	New >450 to ≤480 ms	7/39 (17.9)
	New >480 to ≤500 ms	0/41 (0)
	New >500 ms	0/41 (0)
QT, ms	Increase >30 to ≤60 ms	9/41 (22.0)
	Increase >60 ms	2/41 (4.9)
	New >450 to ≤480 ms	6/39 (15.4)
	New >480 to ≤500 ms	1/41 (2.4)
	New >500 ms	0/41 (0)
PR, ms	Increase >25% and PR >200 ms	1/29 (3.4)
	New PR >200 ms	6/29 (20.7)
QRS, ms	Increase >25% and QRS >120 ms	2/41 (4.9)
	New QRS >120 ms	2/41 (4.9)
Heart rate, bpm	Increase >25% and HR >100 bpm	0/41 (0)
	Decrease >25% and HR <50 bpm	0/41 (0)

*Note*: *n* = number of patients at risk whose worst post‐baseline value met the criterion; *m* = number of patients at risk. ECGs were recorded in triplicate at 0, 4, 8, and 24 h; data are based on values from all ECG assessments.

Abbreviations: bpm, beats per minute; ECG, electrocardiogram; QTcF, absolute QT interval corrected for heart rate by Fridericia's formula.

### 3.9 Laboratory parameters (safety set)

For all haematology parameters analysed, almost all patients had normal values or low‐grade abnormalities (grade 1) at baseline and there were no shifts to higher grades during the study. One patient had grade 3 lymphocyte count decreased (0.43 × 10^9^/L at pre‐dose and 0.35 × 10^9^/L at 24 h post‐infusion).

In terms of blood chemistry, no grade 3/4 abnormalities were observed, with the exception of one patient with a grade 3 serum potassium increase. Worsening of CTCAE grade for serum potassium levels was one of the most frequent post‐baseline abnormalities, irrespective of baseline values; this was reported in 17 patients (41.5%), although only 5 were considered to have clinically relevant changes from baseline (i.e., an AE of hyperkalaemia).

### 3.10 Post‐hoc exploratory analysis (safety set)

Post‐baseline potassium increase (i.e., worsening of baseline CTCAE grade) was observed in 31.6% (*n* = 6/19) of patients with normal renal function (pre‐treatment eGFR: ≥90 mL/min/1.73 m^2^), 37.5% (*n* = 6/16) with mild impairment (≥60–<90 mL/min/1.73 m^2^), and 75.0% (*n* = 3/4) with mild‐to‐moderate impairment (≥45–<60 mL/min/1.73 m^2^). There was no meaningful relationship between post‐baseline potassium increase status and pre‐treatment eGFR score (Pearson correlation coefficient: 0.0373).

## 4 DISCUSSION

Here we present the results of the PASS for LysaKare infusion in patients with GEP‐NET eligible for treatment with ^177^Lu‐DOTATATE, as required as part of the risk‐management plan following EMA approval of LysaKare. Changes in serum potassium following LysaKare infusion were transient, generally peaking at 4 h post‐dose and returning to near baseline within 24 h. Worsening of CTCAE grade for post‐baseline serum potassium levels occurred in 41.5% of patients, including one case of grade 3 increased potassium levels. Additional analyses of potassium levels, other electrolyte levels, and blood gas components mostly showed transient changes that lasted around 24 h, including a general trend towards transient metabolic acidosis. However, the increase in potassium levels and changes in electrolyte and blood gas levels that were suggestive of an acid–base disturbance did not appear to manifest in additional safety issues or clinically significant events, and no AEs of metabolic acidosis were reported. Although most of the AEs reported may have been associated with metabolic acidosis, such as hyperkalaemia, nausea, and vomiting, none were considered serious. In fact, there were no serious AEs or AEs leading to treatment discontinuation/interruption. Overall, six patients (14.6%) had treatment‐related AEs: five had hyperkalaemia (*n* = 1 grade 3) and one had vomiting. However, all patients with hyperkalaemia recovered from the AE within 24 h, either without treatment (*n* = 3) or with furosemide (*n* = 2).

Hyperkalaemia has been reported in other studies investigating the safety of basic amino acid infusions during RLT.^6^, ^10^ Giovacchini et al. reported similar hyperkalaemia findings using the same lysine‐arginine formulation (2.5%) in patients undergoing RLT.^6^ At 4 h post‐dose, 77% (24/31) of patients had serum potassium levels above the normal range (all of whom were clinically asymptomatic), while severe hyperkalaemia (≥6.0 mmol/L) occurred in 19% (6/31) of patients.^6^ By comparison, at 24 h post‐dose, only 13% (4/31) of patients had serum potassium levels above the normal range.^6^ Lapa et al. also reported similar findings with infusion of an L‐arginine and L‐lysine amino acid solution, given at two different doses (2.5% and 1.25%), in patients undergoing RLT.^10^ At 4 h post‐dose, hyperkalaemia (i.e., serum potassium ≥5.0 mmol/L) was observed in 91% (20/22) of patients at both doses, while severe hyperkalaemia (>6.0 mmol/L) was observed in 36% (8/22) of patients at both doses.^10^ However, at 24 h post‐dose, mean serum potassium levels had almost returned to baseline.^10^ Hyperkalaemia incidence and severity were comparable between the two dose cohorts, indicating that varying the dose of an infused amino acid solution did not appear to impact the incidence and severity of hyperkalaemia.^10^

Although there was a trend towards transient metabolic acidosis following LysaKare treatment based on the laboratory values observed in our study, no symptoms of severe metabolic acidosis, such as heart rhythm disturbances, were reported. By contrast, severe metabolic acidosis has been reported in a previous study in which patients received a 2.5% lysine‐arginine amino acid solution prior to RLT.^8^ However, like the LysaKare PASS, patients in the study reported by Pfob et al. typically developed elevated serum potassium (i.e., ≥5.0 mmol/L) 4 h after amino acid infusion.^8^ Despite both studies reporting elevated serum potassium after amino acid infusion, it should be noted that the severe metabolic acidosis reported by Pfob et al. was accompanied by a higher increase in potassium levels 4 h post‐infusion (average increase, 1.66 mmol/L) compared with the present study (mean [SD], 0.60 [0.666] mmol/L). The potassium shift from the intracellular to the extracellular space caused by cationic amino acids has been proposed as a mechanism for hyperkalaemia.^11^, ^17^ However, as suggested by Pfob et al., acidosis may itself decrease potassium secretion and increase reabsorption in collecting ducts, which could be an additional mechanism for hyperkalaemia.^8^ As such, when laboratory values suggestive of metabolic acidosis are observed but are not accompanied by clinically significant events, our findings suggest that clinicians may consider repeating laboratory tests after 24 h to ensure the event is resolving as expected.

Of note, some patients with advanced NET experience electrolyte abnormalities, particularly patients with functioning NET (i.e., NET that are associated with symptoms of hormone hypersecretion).^18^, ^19^, ^20^, ^21^ However, since NET are typically associated with hypokalaemia rather than hyperkalaemia,^18^ functioning NET are unlikely to have been a factor in the development of hyperkalaemia during LysaKare infusion.

The kidneys play a key role in potassium regulation, and impaired renal function can lead to potassium accumulation.^22^ Our post‐hoc exploratory data suggest that similar proportions of patients with normal or mildly impaired renal function at baseline experience increased serum potassium grade following LysaKare infusion, whereas patients with mild‐to‐moderate impairment may be at higher risk. We note that a substantial proportion of patients received concomitant medications that affect potassium homeostasis. Agents such as angiotensin‐converting enzyme inhibitors and angiotensin receptor blockers may have increased potassium levels, whereas medications such as diuretics may have lowered serum potassium levels.^23^ Although these medications may have confounded the observed risk of hyperkalaemia, they were either given to manage pre‐existing medical conditions and were taken throughout the study, including at baseline, or used to treat AEs of hyperkalaemia following a single dose of LysaKare. Therefore, their impact on the observed changes is expected to be minimal. Due to the small sample size, no conclusions can be drawn. Future studies could stratify by renal function to better assess associated risks.

This study has some limitations. First, the primary endpoint analysis included only 25 of the 41 patients treated with LysaKare, as serum potassium measurements were available for this subset; nonetheless, the predefined sample size requirements were still met. Second, LysaKare was administered once, whereas it is indicated for use alongside^177^ Lu‐DOTATATE,^5^ which is administered four times (once every 8 weeks).^1^ As such, it was not possible to assess cumulative effects; however, no cumulative effect is expected, given the posology, nature of the product, and washout period between administrations. Additionally, this study did not investigate outcomes in patient subgroups, such as those with functioning and non‐functioning GEP‐NET; therefore, further investigation is warranted to understand any potential differences in the safety profile of LysaKare between these patient populations.

In conclusion, 41.5% of patients in this study had moderate and transient post‐baseline increases in serum potassium grade. Potassium increase was transient, peaking 4 h after the LysaKare infusion started and spontaneously resolving within 24 h in most cases. Additionally, no new safety signals attributable to LysaKare were identified in this adult population. Per EMA precautions for LysaKare use during RLT with ^177^Lu‐DOTATATE, serum potassium levels should be measured and hyperkalaemia must be corrected before starting LysaKare infusion.^5^ In patients with pre‐existing clinically significant hyperkalaemia, serum potassium levels must be retested before LysaKare infusion to confirm that hyperkalaemia has been corrected.^5^ Patients should be monitored closely for signs of hyperkalaemia, such as dyspnoea, weakness, numbness, chest pain, and cardiac manifestations (conduction abnormalities and cardiac arrythmias).^5^ An ECG should be performed in patients with clinically significant hyperkalaemia before discharge.^5^ All patients should be encouraged to drink substantial quantities of water (at least one glass per hour) on the treatment day to remain hydrated and promote excretion of excess serum potassium.^5^ If hyperkalaemia develops during LysaKare infusion, we recommend administering intravenous furosemide and sodium chloride and retesting serum potassium levels after 24 h.

## AUTHOR CONTRIBUTIONS

**Johannes Hofland:** Investigation, formal analysis, writing—review and editing. **Chiara Maria Grana:** Investigation, formal analysis, writing—review and editing. **Martin O. Weickert:** Investigation, writing—review and editing. **Andrew R. Moore:** Investigation, writing—review and editing. **Tahir Shah:** Investigation, writing—review and editing. **Vineet Prakash:** Investigation, writing—review and editing. **Agnieszka Kolasińska‐Ćwikła:** Investigation, writing—review and editing. **Wouter W. de Herder:** Investigation, writing—review and editing. **Francesca Spada:** Investigation, formal analysis, writing—review and editing. **Lingfei Xu:** Conceptualization, methodology, investigation, formal analysis, writing—review and editing. **Ramon Fite:** Conceptualization, methodology, investigation, formal analysis, writing—review and editing. **Yuan Wu:** Investigation, formal analysis, writing—review and editing. **Jarosław Ćwikła:** Investigation, writing—review and editing. All authors provided final approval of the version to be published and agree to be accountable for the accuracy and integrity of the manuscript.

## FUNDING INFORMATION

This study was funded by Advanced Accelerator Applications, a Novartis company.

## CONFLICT OF INTEREST STATEMENT

Johannes Hofland reports travel or advisory role honoraria from Ipsen, Novartis, and Serb. Chiara Maria Grana reports travel grants and consultancy fees from ITM and Novartis. Martin O. Weickert (retired from September 2025) reports travel grants and NET service support grants from Ipsen and Novartis, and a NET service support grant from Pfizer. Andrew R. Moore has no relevant financial or non‐financial interests to disclose. Tahir Shah reports participation in a joint work project to improve capacity for PRRT at UHB; additionally, as Treasurer of UKINETS, he negotiated sponsorship of the Society's activities by AAA/Novartis. Vineet Prakash has no relevant financial or non‐financial interests to disclose. Agnieszka Kolasińska‐Ćwikła reports consultancy, advisory roles, and honoraria from Ipsen, Novartis, and Merck, and support for travel/accommodation from Ipsen. Wouter W. de Herder reports consultancy, advisory roles, and honoraria from Camarus, Ipsen, and Novartis, and support for travel/accommodation from Ipsen and Recordati. Francesca Spada reports consultancy, educational activities, and travel/accommodation or advisory role honoraria from Ipsen and Novartis/ADACAP. Lingfei Xu, Ramon Fite, and Yuan Wu are employees of Novartis/ADACAP, the Marketing Authorisation Holder of LysaKare. Jarosław Ćwikła reports advisory roles, honoraria, and support for travel/accommodation from Ipsen.

## ETHICS STATEMENT

The trial was performed in accordance with the principles of the Declaration of Helsinki, the International Conference on Harmonisation Good Clinical Practice guidelines, and all applicable regulations. This study was approved by the independent ethics committee for each participating centre (centre number): METC Erasmus MC (3101), Ethics Committee Monzino European Institute of Oncology and Cardiology (3901), NRES Committee West Midlands (4401, 4403, 4404, 4405), Komisja Bioetyczna przy Okręgowej Izbie Lekarskiej w Warszawie (Bioethics Committee at District Medical Chamber Warsaw) (4801).

## INFORMED CONSENT STATEMENT

Informed consent to participate in this study was obtained from eligible patients before any study‐specific procedures were performed.

## Supporting information


**Supplementary Table 1.** Exposure to study treatment (safety set).

## Data Availability

Novartis is committed to sharing with qualified external researchers access to patient‐level data and supporting clinical documents from eligible studies. These requests are reviewed and approved by an independent review panel on the basis of scientific merit. All data provided are anonymized to respect the privacy of patients who have participated in the trial in line with applicable laws and regulations. This trial data availability is according to the criteria and process described on www.clinicalstudydatarequest.com.
