# Supplementary material for: The effect of LysaKare infusion on serum potassium levels in patients with gastroenteropancreatic neuroendocrine tumours eligible for treatment with [ 177Lu]Lu‐DOTA‐TATE: A post‐authorisation safety study
Source: J Neuroendocrinol. 2026 Apr 13;38(4):e70172. doi: 10.1111/jne.70172 (PMC13077017; doi:10.1111/jne.70172)
Supplement: Supplementary file 1 — Supplementary Table 1. Exposure to study treatment (safety set). [file JNE-38-e70172-s001.docx]

# Supplementary materials

**The effect of LysaKare infusion on serum potassium levels in patients with gastroenteropancreatic neuroendocrine tumours eligible for treatment with
[^177^Lu]Lu-DOTA-TATE: a post-authorisation safety study**

*Journal of Neuroendocrinology*

Johannes Hofland, Chiara Maria Grana, Martin O. Weickert, Andrew R. Moore, Tahir Shah,
Vineet Prakash, Agnieszka Kolasińska-Ćwikła, Wouter W. de Herder, Francesca Spada, Lingfei Xu, Ramon Fite, Yuan Wu, Jarosław B. Ćwikła

**Corresponding author:**
Dr Johannes Hofland
Department of Internal Medicine, Section of Endocrinology, ENETS Center of Excellence, Erasmus MC Cancer Institute, Rotterdam, Netherlands
[j.hofland@erasmusmc.nl](mailto:j.hofland@erasmusmc.nl)

**Supplementary Table 1** Exposure to study treatment (safety set)

|  | All patients (*N* = 41) |
| --- | --- |
| Duration of exposure, hours |  |
| Median | 4.00 |
| Q1–Q3 | 4.00–4.03 |
| Range | 3.67–4.28 |
| Duration of exposure categories, no. (%) |  |
| 2 to <3.67^a^ hours | 1 (2.4) |
| ≥3.67 to <4.33 hours | 40 (97.6) |
| ≥4.33 hours | 0 |
| Actual total volume, mL |  |
| Mean (SD) | 999.8 (1.56) |
| Median | 1000.0 |
| Range | 990–1000 |

Abbreviations: Q, quartile; SD, standard deviation.

^a^One patient with 3 hour 40 minutes = 3.667 hours.
